# Imagined and Actual Acupuncture Effects on Chronic Low Back Pain: A Preliminary Study

**DOI:** 10.1155/2020/8579743

**Published:** 2020-07-01

**Authors:** Jin Cao, Scott P. Orr, Georgia Wilson, Jian Kong

**Affiliations:** Department of Psychiatry, Massachusetts General Hospital, Harvard Medical School, Charlestown, 02129 MA, USA

## Abstract

**Background:**

Research suggests that imagined experiences can produce brain responses similar to those produced by actual experiences. Shared brain responses that support both imagination and perception may underlie the functional nature of mental imagery. In a previous study, we combined acupuncture and imagery to develop a new treatment method, video-guided acupuncture imagery treatment (VGAIT). We found that VGAIT significantly increased pain thresholds in healthy subjects. The aim of this study is to extend our previous finding by investigating whether VGAIT can relieve symptoms in patients with chronic low back pain.

**Methods:**

We first performed a single-arm study in which we administered video-guided acupuncture imagery treatment (VGAIT) on patients with chronic low back pain (cLBP) (Study 1, *n* = 18, 12 females). We then compared our findings to those from a recently published study in which real or sham acupuncture treatment was applied on patients with cLBP (Study 2, *n* = 50, 31 females) using a similar protocol. All patients in Studies 1 and 2 received 6 treatments over 4 weeks.

**Results:**

All three treatments (VGAIT, real, and sham acupuncture) significantly reduced pain severity as measured by a low back pain bothersomeness score. VGAIT produced similar effects to real acupuncture (*p* = 0.97) and nonsignificantly greater pain bothersomeness relief compared to sham acupuncture (*p* = 0.14). Additional analysis showed that there was no significant difference on the sensations evoked by different treatment modalities.

**Conclusion:**

These findings support VGAIT as a promising method for pain management.

## 1. Introduction

Chronic low back pain (cLBP) is a highly prevalent and disabling disorder with few satisfactory treatment options [1, 2]. Opioids are the most commonly prescribed class of drugs for cLBP [3], but the misuse of opioids has emerged as a serious substance abuse crisis. Thus, there is an urgent need for effective, nonopioid treatments for chronic pain [4, 5].

Both acupuncture and imagery have long been used in medical practice, including the treatment of chronic pain [6–9]. However, the mechanisms that mediate acupuncture and imagery efficacy remain poorly understood. Neuroimaging studies have found that acupuncture needle manipulation can produce wide-spread brain activations and deactivations [10–14]. Studies have also found that imagined experiences can produce brain responses similar to those that occur during actual experiences [15–17]. Thus, shared neurocognitive responses that support both imagination and perception may explain the functional nature of mental imagery [16, 18].

Accumulating evidence suggests that anticipation, attention, and belief of acupuncture needle stimulation may also produce wide-spread brain activity/connectivity changes. In an early study [14], Jung et al. found that both genuine and pseudostimulation resulted in brain activations in the insula, anterior cingulate cortex, secondary somatosensory cortex, superior parietal cortex, and brain deactivation in the medial prefrontal cortex, posterior cingulate cortex, inferior parietal cortex, and parahippocampus. In another study [19], they found that cutaneous electrical stimuli without actual stimulation on acupoints resulted in greater de qi sensation compared to the control condition. Cognitive components of cutaneous electrical stimulation are associated with brain activation in the anterior insula, presupplementary motor area, and secondary somatosensory area. The expectations of acupuncture stimulation resulted in distinct experiences of somatosensation as well as brain activations in the insula and presupplementary motor area.

Makary et al. [13] investigated the effect of a specific form of sham acupuncture (phantom acupuncture (PHNT)) that reproduces the acupuncture needling procedure without somatosensory tactile stimulation. They found that PHNT can produce bilateral activation in the primary and secondary somatosensory cortex in patients with low back pain. In addition, the patients reported vicarious acupuncture sensations without needling stimulation. In a later study [20], they found that reduced low back pain from PHNT was negatively correlated with increased posterior cingulate cortex–anterior insula connectivity and exhibited a trend towards positive correlation with decreased primary somatosensory– (S1–) posterior insula connectivity.

In a previous study, we combined acupuncture and imagery to develop a new treatment method, video-guided acupuncture imagery treatment (VGAIT). During VGAIT, the participants watch a video of acupuncture that has been previously administered to their body and imagine it being concurrently applied. We found that VGAIT significantly increases pain thresholds in healthy subjects. In addition, we found that brain activity in insula and rostral anterior cingulate cortex (rACC), key regions in pain processing and modulation, was associated with analgesia evoked by both real acupuncture and VGAIT respectively [21].

The present pilot study aimed to extend our previous finding by investigating whether VGAIT can relieve symptoms in patients with cLBP. Specifically, we first performed a single-arm study in which cLBP patients received 6 sessions of VGAIT over 4 weeks (Study 1). Next, we compared the findings from Study 1 with those from a recently published study (Study 2) by our group that investigated the treatment effect of acupuncture and context in cLBP subjects [22]. Both studies used the same treatment frequencies and clinical outcome measures.

## 2. Methods

### 2.1. Study 1: Single-Arm trial on the Effect of VGAIT in cLBP Patients

Twenty cLBP patients were recruited. All patients were allowed to continue their existing medication and treatments. The study was approved by the Partners Human Research Committee/IRB (Institutional Review Board of Massachusetts General Hospital). All subjects provided signed informed consent before starting the study.

Patients were eligible for participation if they (1) were 18 to 60 years of age and met the Classification Criteria for cLBP (having low back pain for more than 6 months), (2) were without other severe chronic pain comorbidities, (3) scored at least 4 on the 11-point (0-10) LBP severity scale, and (4) had a prior evaluation of their low back pain by a health care provider. Patients were excluded if there was / were (1) a specific cause of back pain (e.g., cancer, fractures, and infections), (2) complicated back problems (e.g., prior back surgery and medico legal issues), (3) possible contraindications for acupuncture (e.g., coagulation disorders, cardiac pacemakers, pregnancy, and seizure disorder) and conditions that might confound longitudinal effects or interpretation of results (e.g., severe fibromyalgia and rheumatoid arthritis), (4) conditions making study participation difficult (e.g., paralysis, psychoses, or other severe psychological problems as per the judgment of a study investigator), (5) intent to undergo surgery during the time of involvement in the study, (6) active substance abuse disorder in the past 24 months, as determined by self-report and/or urine toxicology, and (7) inability to provide informed consent for oneself.

Subjects participated in 8 experimental sessions. Session 1 was a baseline assessment during which subjects received acupuncture exposure for about 5 minutes that was video-recorded for use in following treatment sessions. A modified standardized acupuncture protocol based on previous cLBP clinical trials was used [23]. This treatment is considered effective by cLBP experts [24]. The 7 real acupoints used were Yaoyangguan (GV3), bilateral Shenshu (BL23), bilateral Weizhong (BL40), bilateral Taixi (KI3), and 1-3 ashi (tender) points bilaterally on the lower back and legs. The rationale for the selection of these acupoints has been published in a previous clinical trial protocol on acupuncture treatment of low back pain [24]. A licensed acupuncturist performed the acupuncture exposure. Detailed selection of acupoints for acupuncture exposure can be found in Supplementary 1.

**Figure 1:**
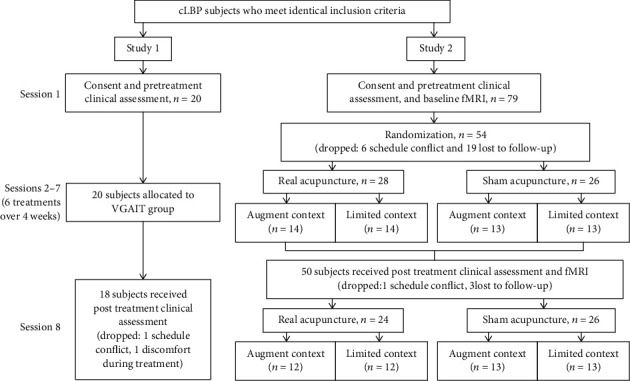
Study design and conduct details. cLBP: chronic low back pain.

Sessions 2-7 were treatment sessions, during which subjects received VGAIT. The time between Session 1 and Session 2 ranged from 1 to 7 days. At the beginning of the VGAIT session, the subjects first read a script introducing the imagery acupuncture treatment along the following lines: “First you will watch a one-minute fixation video. As you watch, relax and concentrate on your breathing. You will then see a video of acupuncture treatment being applied on your low back and leg for about 25 minutes. Focus on the needle manipulation and try to imagine there is an actual needle being placed into your low back and leg at the same spot. You may feel some soreness and an aching, dull pain, along with other sensations. You will find that you can actually feel the needle manipulation at the same spot as in the video. The more vivid and real the sensation, the more effective the treatment, so it is very important that you stay focused and try to imagine the sensation of receiving acupuncture.” Session 8 was the posttreatment assessment, during which clinical assessments (identical to Session 1) were conducted (Figure 1).

#### 2.1.1. Study Intervention

All subjects received 6 treatments over 4 weeks (twice weekly for the first 2 weeks, then once weekly for the last 2 weeks), and each VGAIT treatment lasted about 25 minutes. During VGAIT, the acupuncture needles were rotated at one acupoint and then another in 10-second rotations with 15-second breaks in between, and about 1 minute breaks after each round. Each acupoint was stimulated 4 times.

#### 2.1.2. Outcome Measures

The primary outcome was a measure of how bothersome one's low back pain (LBP) was during the past week, rated on an 11-point visual analogue scale of 0 (“not at all bothersome”) to 10 (“extremely bothersome”) [23]. Secondary outcome measures included 8 subdomains (pain interference, depression, physical function, fatigue, anxiety, sleep disturbance, social disability, and pain intensity) that evaluated how chronic pain diminished each patient's quality of life [25]. These measures were assessed using the Patient Reported Outcomes Measurement Information System (PROMIS-29). In addition, given that different intensities of sensations from treatment may be a potential factor that influences clinical outcome, the Massachusetts General Hospital Acupuncture Sensation Scale (MASS) [26, 27] was administered to subjects in each session.

### 2.2. Study 2: A Randomized Clinical Trial Investigating the Effect of Acupuncture and Context in Patients with cLBP

Procedures for Study 2 were similar to those of Study 1, with the exception that subjects in Study 2 participated in 2 MRI scans before and after the 6 treatment sessions. Please see the original publication for details of the experiment [22]. In summary, seventy-nine subjects with cLBP were recruited into this study using the criteria identical to those of Study 1 (Figure 1).

Subjects were randomized into 4 groups (real or sham acupuncture by augmented or limited context groups). Previous studies have suggested that expectancy/context may modulate acupuncture treatment response [28, 29]. Thus, we also attempted to evaluate the context effect using a context manipulation model (Figure 1) [29]. During data acquisition, all study personnel except the acupuncturist were blinded with respect to the intervention condition. Subjects were also blinded as to whether they were receiving real or sham acupuncture. At the end of the study, an investigator debriefed the participant and explained the reason for maintaining intervention blindness.

#### 2.2.1. Study Interventions

The acupoints used for real acupuncture exposure were identical to those used in Study 1. All treatments were performed by licensed acupuncturists. Each treatment lasted 25 minutes, with additional stimulation applied to elicit “de qi” by twirling the needles at 10 minutes and again just prior to needle removal.

Sham acupuncture was applied at 12 sham points with a Streitberger needle. Instead of penetrating the skin, the point of the Streitberger needle retracts up the handle shaft when the acupuncturist presses it against the skin. This sham device has been validated by studies showing that subjects cannot distinguish between real and sham needling [30, 31]. A detailed description of acupoints and sham points for real and sham acupuncture treatments can be found in Supplementary 1.

#### 2.2.2. Outcome Measures

Outcome measures identical to those of Study 1 were applied, allowing comparisons to be made between the 2 studies.

### 2.3. Statistical Analyses

For demographic characteristics, a Chi-square test and one-way ANOVA were applied to assess gender and age differences between groups. For clinical outcomes, a paired sampled *t*-test was performed for within-group analyses (pre- vs. posttreatment), and a one-way ANOVA was performed for between-group analyses (real vs. sham, sham vs. VGAIT, and real vs. VGAIT). All analyses were conducted using the R program incorporated in the JASP software (Version 0.8.1, http://www.jasp-stats.org). All statistical tests were 2-tailed, and *p* values of <0.05 were considered to be statistically significant.

## 3. Results

Of the 20 subjects recruited for Study 1, 18 completed the study (1 subject dropped out due to scheduling conflicts and 1 subject dropped out due to discomfort while watching their VGAIT video recording). Of the 79 subjects recruited for Study 2, 50 completed the study (please see Figure 1 for subject drop out reasons). A total of 68 subjects (43 females; age 39.87 ± 1.57 years, mean ± SE) were included in analyses (*n* = 18, 24, and 26 for VGAIT, real acupuncture, and sham acupuncture groups, respectively). No significant difference across the 3 treatment groups was found for age (*F*_(2, 65)_ = 0.10, *p* = 0.91) and gender (*χ*^2^ = 0.56, *p* = 0.76) (Table 1). Study design and conduct details are shown in Figure 1.

### 3.1. Primary Outcome

#### 3.1.1. Study 1

LBP bothersomeness ratings were significantly reduced after 6 VGAIT sessions compared to pretreatment ratings (2.94 ± 0.41, *t*_(17)_ = 7.08, *p* < 0.001).

#### 3.1.2. Study 2

LBP bothersomeness ratings were significantly reduced after the 6 treatments for all 4 groups. A two-way analysis of covariance (ANCOVA) with treatment (real vs. sham) and context (augment vs. limited) as factors indicated no significant difference between the augmented and limited context groups (*F*_(1, 48)_ = 0.75, *p* = 0.39) but detected a marginally significant difference between real and sham acupuncture (*F*_(1, 48)_ = 3.06, *p* = 0.09). Thus, the 4 groups were combined into 2 groups (real and sham acupuncture) for the following analyses. A detailed description of statistical analyses can be found in our previously published paper [22]. The pre- vs. posttreatment changes for LBP bothersomeness in Studies 1 and 2, as well as changes in each participant's LBP bothersomeness rating before and after treatment, can be found in Table 1 and Supplementary 2, respectively.

**Figure 2:**
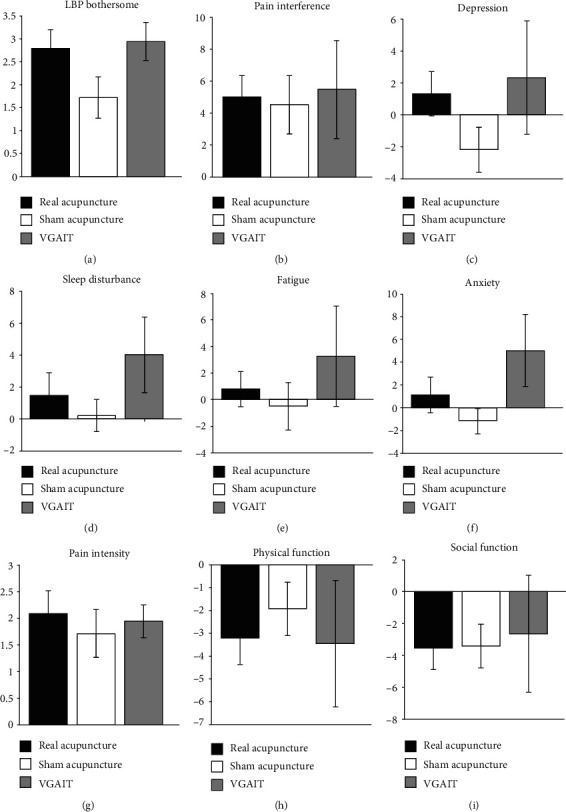
Clinical outcome changes (pre- minus post-treatment). Primary outcome: (a) LBP bothersomeness change. Secondary outcomes: (b–i) PROMIS-29 subdomain changes. VGAIT: video-guided imagery acupuncture treatment.

Comparison between VGAIT (Study 1) and real and sham acupuncture (Study 2) using ANOVA revealed a trend towards significance among the 3 groups (bothersomeness: *F*_(2, 65)_ = 2.47, *p* = 0.09). Post hoc analysis (Tukey's correction) showed that compared with sham acupuncture, VGAIT produced a nonsignificant LBP bothersomeness reduction (*t* = −1.94, *p* = 0.14). There was a comparable LBP bothersomeness reduction between real acupuncture and VGAIT (*t* = −0.24, *p* = 0.97) (Figure 2(a)).

### 3.2. Secondary Outcomes

Analyses of the PROMIS-29 subdomains showed that after 6 VGAIT sessions, the subjects reported significant pain intensity reduction during the past week (*t* = 6.32, *p* < 0.001).

In Study 2, the subjects in the real acupuncture group showed significant improvements in pain interference, pain intensity, physical function, and social function (*t* = 3.82, *p* < 0.001; *t* = 4.71, *p* < 0.001; *t* = −2.75, *p* = 0.01; and *t* = −2.70, *p* = 0.01, respectively) following the treatments. Twenty-four subjects in the sham acupuncture group completed the PROMIS questionnaire and reported significant improvements in pain interference, pain intensity, and social function (*t* = 2.38, *p* = 0.03; *t* = 3.65, *p* < 0.001; and *t* = −2.39, *p* = 0.03, respectively). Detailed pre- vs. posttreatment changes for each PROMIS-29 subdomain in Studies 1 and 2 can be found in Table 1.

Between-group analyses of pre- and posttreatment changes across the 3 groups using ANOVA revealed no significant difference among the 3 groups. However, we observed a trend for anxiety (*F*_(2, 63)_ = 2.39, *p* = 0.10). Post hoc analysis (Tukey's correction) showed that compared with sham acupuncture, VGAIT demonstrated a marginally significant greater reduction in anxiety, as measured by PROMIS-29 (*t* = −2.18, *p* = 0.08) (Figures 2(b)–2(i)).

### 3.3. MASS Ratings

**Figure 3:**
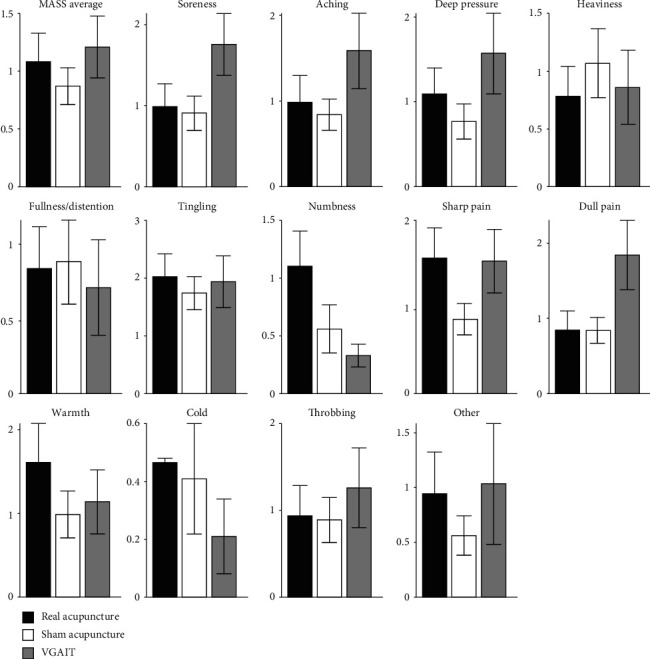
MASS average sensation ratings evoked by different treatment modalities. MASS: Massachusetts General Hospital Acupuncture Sensation Scale; VGAIT: video-guided acupuncture imagery treatment.

We also administered the MASS to measure sensations evoked by VGAIT, real acupuncture, and sham acupuncture after each treatment. The average sensation ratings evoked by the 3 treatment modalities are shown in Figure 3. Further analysis showed no significant association between the MASS score and bothersomeness rating changes (*p* values ranged from 0.09 to 0.77 for different MASS sensations) in the VGAIT group. ANOVA showed no significant difference in average MASS score across the 3 treatment modalities (*F*_(2, 65)_ = 0.58, *p* = 0.56).

## 4. Discussion

The reported work examined the treatment effect of a novel pain management method that combines acupuncture with video-guided imagery. Our preliminary results, when compared with results from a previous study [22], showed that VGAIT produced similar effects as real acupuncture and a non-significant greater reduction in pain severity compared to sham acupuncture. These results support the potential of VGAIT as a novel pain management method.

Both acupuncture [20, 32–34] and guided imagery treatment [7, 35, 36] have demonstrated efficacy as methods for pain management. We found that VGAIT, a combination of the two methods, not only relieved pain bothersomeness in patients with cLBP but also produced a marginally significant greater reduction in anxiety level compared to sham acupuncture treatment. Accumulating evidence suggests that chronic pain conditions are comorbid with psychological distress, especially anxiety [37]. Anxiety may play an important role in perpetuating the distress associated with cLBP, as persistent pain and limited physical movement likely impact the brain circuitry that processes emotion. This increased distress, in turn, aggravates anxiety and worsens pain perception [38].

The mechanism by which VGAIT reduces back pain is unclear. In a previous study, we found that VGAIT can produce greater fMRI signal decreases at the rACC in healthy subjects [21]. The rACC is a key region in the descending pain modulation system (DPMS) [39, 40]. Previous findings have suggested that the rACC plays an important role in the pathophysiology of cLBP. In addition, the ACC is a key region for interoception, which encompasses the integration of signals relayed from the body to the brain. The ACC plays an important role in maintaining the body's homeostatic conditions [41] and potentially aids in self-awareness [42]. Taken together, we speculate that VGAIT may produce its beneficial effect through the ACC and the ACC's associated pain modulation and interoception processes. Nevertheless, this hypothesis needs to be tested in future work.

Similar to our previous study [21], we found that VGAIT also produced sensations associated with acupuncture treatment. There was no significant difference among the three treatment modalities. The guided imagery used in VGAIT may help participants recall previous acupuncture experience and sensations. It may also induce a more goal-directed state in which patients can work towards alleviation of pain severity.

The reported findings must be viewed as preliminary, as there was no control condition in Study 1. We believe that a VGAIT control condition would produce results similar to those of sham acupuncture (Study 2); however, a randomized study that includes a VGAIT control condition is clearly needed. Also, the participants in Study 1 were recruited separately from those in Study 2. Thus, different patient consent forms were used. Patients in Study 2 also knew that they would receive real or sham acupuncture, while patients in Study 1 knew that they would receive VGAIT. Therefore, psychological characteristics of patients in Studies 1 and 2 may have differed. A follow-up session that assesses the persistence of pain alleviation should be included in future studies to evaluate the long-term effects of VGAIT and acupuncture. Finally, objective measurements such as those obtained from neuroimaging are needed to explore the potential mechanisms underlying VGAIT and acupuncture.

## 5. Conclusions

In summary, our preliminary results suggest that VGAIT has potential as a treatment for chronic low back pain that optimizes time, cost, and available resources. VGAIT may be considered a therapeutic option in the multidisciplinary management of chronic pain that can be combined with other treatments or independently administered for pain relief.

## Figures and Tables

**Table 1:** Demographics and clinical outcome changes for within-group analyses.

Group	Study 1: VGAIT	Study 2: Real	Study 2: Sham
Demographics			
*N*	18	24	26
Gender	12 females	16 females	15 females
Age (years)	40.78 ± 3.05	39.00 ± 2.56	40.04 ± 2.69
Clinical outcome changes (pre–post) in within-group analyses			
*N*	18	24	26
LBP bothersomeness	2.94 ± 0.42^∗∗^	2.79 ± 0.41^∗∗^	1.72 ± 0.45^∗∗^
*N*	18	24	24
PROMIS-pain interference	5.48 ± 3.07	5.00 ± 1.31^∗∗^	4.53 ± 1.90^∗^
PROMIS-depression	2.34 ± 3.55	1.33 ± 1.37	−2.17 ± 1.47
PROMIS-sleep disturbance	4.04 ± 2.35	1.48 ± 1.43	0.22 ± 1.04
PROMIS-fatigue	3.27 ± 3.76	0.80 ± 1.31	−0.50 ± 1.86
PROMIS-anxiety	5.02 ± 3.16	1.13 ± 1.57	−1.14 ± 1.17
PROMIS-pain intensity	1.94 ± 0.31^∗∗^	2.08 ± 0.44^∗∗^	1.71 ± 0.47^∗∗^
PROMIS- physical function	−3.45 ± 2.76	−3.21 ± 1.17^∗^	−1.93 ± 1.22
PROMIS-social function	−2.64 ± 3.66	−3.55 ± 1.31^∗^	−3.40 ± 1.42^∗^

Results presented are mean ± SE. Changes reflect pre- minus post-treatment scores. “^∗^” and “^∗∗^” identify *p* < 0.05 and *p* < 0.001, respectively, for pre- vs. posttreatment within-group comparisons. VGAIT: video-guided acupuncture imagery treatment; Real: real acupuncture; Sham: sham acupuncture.

## Data Availability

Data used to support the findings of this study are available from the corresponding author upon request.

## Supplementary Materials

Supplementary Materials Supplementary 1: Acupoint/sham point descriptions for real and sham acupuncture treatment. Supplementary 2: Changs (pre- minus post-treatment) in each patient's LBP bothersomeness rating before and after treatment.
