# Supplementary material for: Imagined and Actual Acupuncture Effects on Chronic Low Back Pain: A Preliminary Study
Source: Neural Plast. 2020 Jul 1;2020:8579743. doi: 10.1155/2020/8579743 (PMC7350074; doi:10.1155/2020/8579743)
Supplement: Supplementary Materials — Supplementary 1: Acupoint/sham point descriptions for real and sham acupuncture treatment. Supplementary 2: Changs (pre- minus post-treatment) in each patient's LBP bothersomeness rating before and after treatment. [file 8579743.f1.docx]

Supplementary Table 1: Acupoint / sham point descriptions for real and sham acupuncture treatment

| ***Acupoints for cLBP: 7 regular acupoints plus 1 to 3 ashi (tender) points*** | | | |
| --- | --- | --- | --- |
| GV 3: | *In the depression below the spinous process of the 4th lumbar vertebra.* | BL23: | *1.5 cun lateral to the lower border of the spinous process of the second lumbar vertebra.* |
| BL 40: | *Midpoint of the transverse crease of the popliteal crease.* | KI-3: | *Depression between the medial malleolus and the tendocalcaneus level with the vertex of the medial malleolus.* |
| ***Sham Points for cLBP: 12 points in total*** | | | |
| SH 1-8: | *About 5 cun lateral from the lateral bladder meridian on both sides (bilateral with each side 4 points) and level with BL23-26 ** | SH 9-10: | *Two non-acupuncture points on the thigh: about 3 cun medially and 3 cun caudally from BL 37 (bilateral)* |
| SH 11-12: | *About 1 cun inferior and 1 cun medial to BL57 (bilateral)*** |  |  |

Notes: cLBP, chronic low back pain; GV, the Governing Vessel; BL, the Bladder meridian; KI, the Kidney meridian; SH, sham point; TCM, Traditional Chinese Medicine. 1 cun is about 2 cm; it is a measurement relative to the body dimensions of each patient, defined by conventional TCM guidelines.


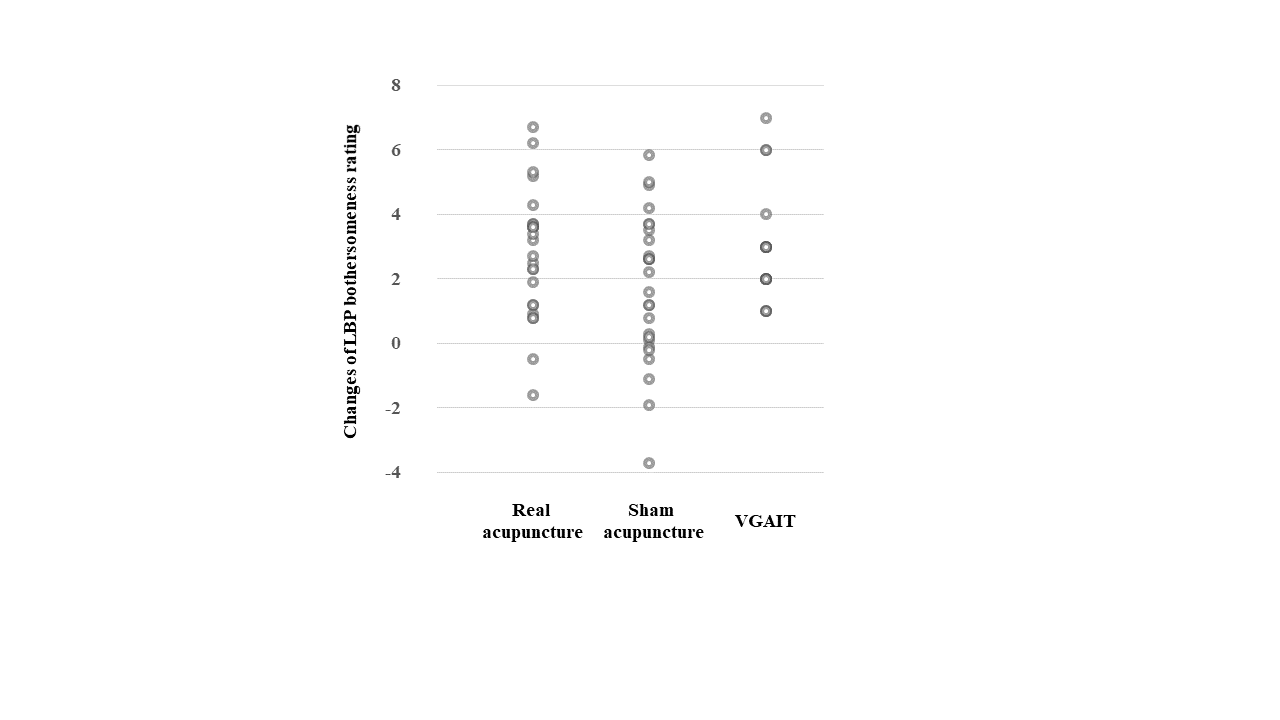


Supplementary Figure 1: Changes (pre- minus post-treatment) in each patient’s LBP bothersomeness rating before and after treatment. Abbreviations: LBP, low back pain; VGAIT, video-guided acupuncture imagery treatment.
